# Dysplasia and Colorectal Cancer Surveillance in Ulcerative Colitis Patients in Latin America: Real-World Data

**DOI:** 10.1093/crocol/otae081

**Published:** 2025-01-14

**Authors:** Viviana Parra-Izquierdo, William Otero-Regino, Fabian Juliao-Baños, Juan Sebastián Frías-Ordoñez, Edgar Ibañez-Pinilla, Fabio Leonel Gil-Parada, Hernando Marulanda-Fernández, Lina Otero-Parra, Elder Otero-Ramos, Fabian Eduardo Puentes-Manosalva, Gerardo Andrés Guzmán Rojas, Kenneth Ernest-Suárez, Keyla Villa-Ovalles, Juan Eloy Paredes-Mendez, María Luisa Jara-Alba, David Andrade-Zamora, Manuel Alonso Ardila-Báez, Cristian Flórez-Sarmiento, Guillermo Veitia, Abel Sánchez, Lazaro Antonio Arango-Molano, Fernando Fluxa, Natália Sousa Freitas Queiroz, Mariastella Serrano

**Affiliations:** Gastroenterology and Rheumatology, International Hospital of Colombia, Bucaramanga, Colombia; Cellular and Molecular Immunology Group (INMUBO), Universidad El Bosque, Bogotá, Colombia; Gastroenterology, National University of Colombia, Bogota, Colombia; Gastroenterology and Digestive Endoscopy, National University Hospital of Colombia, Bogota, Colombia; Gastroenterology and Digestive Endoscopy, Gastroenterology and Endoscopy Center, Bogota, Colombia; Gastroenterology, Pablo Tobón Uribe Hospital, Medellín, Colombia; Gastroenterology, National University of Colombia, Bogota, Colombia; Gastroenterology and Digestive Endoscopy, National University Hospital of Colombia, Bogota, Colombia; Faculty of Medicine, El Bosque University, Bogota, Colombia; Colombia Clinic, Bogota, Colombia; Gastroenterology, National University of Colombia, Bogota, Colombia; Gastroenterology and Digestive Endoscopy, National University Hospital of Colombia, Bogota, Colombia; Gastroenterology and Digestive Endoscopy, Gastroenterology and Endoscopy Center, Bogota, Colombia; Central Police Hospital, Bogota, Colombia; Gastroenterology, National University of Colombia, Bogota, Colombia; Gastroenterology and Digestive Endoscopy, Gastroenterology and Endoscopy Center, Bogota, Colombia; Gastroenterology and Digestive Endoscopy, Gastroenterology and Endoscopy Center, Bogota, Colombia; Central Police Hospital, Bogota, Colombia; Gastroenterology, University of Caldas, Union of Surgeons, ODO, Zentria Salud. Manizales, Caldas, Colombia; Gastroenterology, Farallones Clinic, Cali, Valle del Cauca, Colombia; Gastroenterology, Colsanitas Chipi Chape Medical Center Cali, Valle del Cauca, Colombia; School of Medicine, University of Costa Rica, San José, Costa Rica; Inflammatory Bowel Disease Unit, Hospital México, Caja Costarricense de Seguro Social, San José, Costa Rica; Gastroenterology and Digestive Endoscopy, Hospital Luis E Aybar, Santo Domingo, Dominican Republic; Gastroenterology, Guillermo Almenara National Hospital, Lima, Perú; Gastroenterology, International Clinic, Lima, Peru; Gastroenterology, Teodoro Maldonado Carbo Specialty Hospital, Guayaquil, Ecuador; Gastroenterology, Hospital of the Ecuadorian Institute of Social Security of Cuenca, Cuenca, Ecuador; Gastroenterology, University of La Sabana, Chía, Colombia; Cellular and Molecular Immunology Group (INMUBO), Universidad El Bosque, Bogotá, Colombia; Gastroenterology, Hospital Internacional de Colombia, Bucaramanga, Colombia; Gastroenterology, Hospital Vargas de Caracas, Caracas, Venezuela; Gastroenterology, Universidad Central de Venezuela, Caracas, Venezuela; Gastroenterology and Digestive Endoscopy, Roosevelt Hospital, Guatemala City. Guatemala; Gastroenterology, University of Caldas, Union of Surgeons, ODO, Zentria Salud. Manizales, Caldas, Colombia; Gastroenterology, Clínica Meds, Santiago, Chile; Health Sciences Graduate Program, Pontifícia Universidade Católica do Paraná (PUCPR), Curitiba, Brazil; Department of Pediatrics, Georgetown University, Washington DC, USA

**Keywords:** (**MeSH):** inflammatory bowel diseases, colonoscopy, colonic neoplasms, prognosis, colitis, ulcerative

## Abstract

**Background:**

The prevalence of colorectal cancer (CRC) in patients with ulcerative colitis (UC) is higher than in the general population, in Latin America there is a progressive increase of UC, and information about CRC screening in inflammatory bowel disease (IBD) is scarce. The aim of this study was to analyze the findings of endoscopic surveillance of CRC in patients with IBD according to available technology.

**Methods:**

Multicenter, cross-sectional, analytical study conducted in Latin American countries, in patients with UC, predominantly with more than 8 years of diagnosis and different degrees of disease activity. Surveillance colonoscopies were performed according to available technology. Risk factors for dysplasia detection were analyzed.

**Results:**

One hundred and forty-four patients, 55.5% women, mean age 47.3 (range 17.1 to 90; SD 15.64) years and mean duration of disease 12.71 (range 0.64 to 57.13; SD 8.08) years. Forty-nine lesions were identified, 18 corresponded to dysplasia. The detection rate of dysplasia per lesion and per procedure was 36.7% and 12.5%, respectively. By logistic regression analysis, the duration of disease (OR 1.12;95%CI:1.047 to 1.215, *P* = .002) and the presence of post-inflammatory polyps (OR 3.4;95%CI:1.11 to 10.36, *P* = .031) were risk factors for higher detection of dysplasia. Digital chromoendoscopy was associated with greater detection of dysplasia (OR 4.99, 95%CI: 1.092 to 22.864, *P* = .038).

**Conclusions:**

In our region, the duration of disease and the presence of post-inflammatory polyps were the factors with the highest association for dysplasia detection, and digital chromoendoscopy with directed biopsies was the technique of choice. The implementation of a specific surveillance program in colonoscopy in IBD is an effective strategy to achieve high detection rates.

## Graphical Abstract

**Graphical Abstract:**
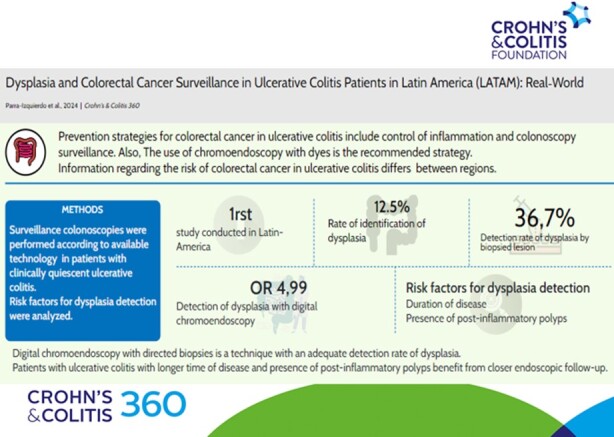

## Introduction

Ulcerative colitis (UC) along with Crohn’s disease (CD) represents the most frequent diagnosis in patients with inflammatory bowel disease (IBD).^1^ Unlike CD, which can involve any part of the digestive tract from the mouth to the perianal area, UC only affects the colon and occasionally the distal ileum.^2^ Traditionally, inflammation in UC has been considered to be limited to the mucous layer.^3^ However, depending on the severity and chronicity, it can produce fibrosis of the submucosa and thickening of the muscle.^4^ It usually starts in the rectum, and can progressively spread proximally to the cecum.^5^ Morbidity and mortality increase in relation to the extent of colonic involvement.^6^ The risk of CRC in UC is 2.4 times that of the general population.^7^ The main risk factors for CRC are extensive disease, evolution of 8 to 10 years, young age at diagnosis, male sex, family history of CRC, stenosis of the colon, primary sclerosing cholangitis, post-inflammatory polyps (“pseudopolyps”), although there is controversy about these^8,9^ and the presence of dysplasia.^10^ The risk increases from 0.5% to 1% one year after UC diagnosis, with estimated incidences of 1.6% at 10 years, 8.3% at 20 years, and 18.4% at 30 years after the onset of UC.^11^ However, in the last 20 years, a sustained decline in the incidence of CRC has been observed, probably due to better control of inflammation using available biologic therapy, as well as the use of 5-aminosalicylates (5-ASA),^12^ which appears to have intrinsic antineoplastic properties.^13^ Although the mechanisms responsible for CRC in IBD are not currently known, DNA damage, secondary to oxidative stress derived from chronic inflammation,^14^ as well as factors secondary to the host immune response with the participation of metabolites derived from the gut microbiome^14^ appear to be contributory factors. This CRC associated with colitis would follow the following sequence: chronic colitis—low-grade dysplasia—high-grade dysplasia—carcinoma. In contrast, sporadic CRC follows the adenoma-carcinoma sequence.^14^ Current strategies to prevent CRC primarily include sustained and profound control of inflammation and colonoscopy surveillance, which has been shown to be protective against the development of advanced CRC in these patients^15,16^ and has been recommended by scientific societies and experts worldwide. It is recommended to complete this after 8 years of disease extending beyond the rectum.^14,17,18^ In a recent systematic review, CRC detection rates were significantly higher in the group under no endoscopic surveillance follow-up (135/4,256, 3.2%) compared to the follow-up group (53/2,895, 1.8%; OR: 0.58; 95% CI: 0.42 to 0.80; *P* < .001. The objective of surveillance is to identify lesions in early stages to avoid advanced cancers and thus mortality.^19^ In 2015, the consensus on dysplasia screening in IBD “SCENIC” (“Surveillance for Colorectal Endoscopic Neoplasia Detection and Management in IBD Patients: International Consensus Recommendations”) was published, recommending targeted biopsies with chromoendoscopy,^11^ in order to detect malignant or premalignant lesions.^20^ When primary sclerosing cholangitis is present, surveillance is recommended from the first year of disease regardless of the activity, extent, and duration of UC, and when there are other risk factors, surveillance is personalized in accordance.^10^ Most dysplasias are endoscopically visible, which makes the performance of targeted biopsies superior to random biopsies.^20^ The use of chromoendoscopy with dyes^17^ to identify and characterize is the recommended strategy, as well as digital chromoendoscopy.^20–22^

In Latin America (LATAM) there are no studies on the surveillance and performance of these to detect dysplasia or CRC in patients with UC. Additionally, some of the data on this population and pertaining to cancer risk may not be applicable to Latin American patients considering the differences in genetics, environment, and microbiome between this population and other groups of patients with IBD in Europe, Asia, and North America. Available technologies in this region, include chromoendoscopy with dyes and digital chromoendoscopy, which have shown utility in real-life studies.^18,21,22^ Considering the above, we decided to carry out the present international multicenter study. The objectives were to determine the Latin American prevalence of dysplasia or CRC in patients with UC, describe the risk factors for neoplasia in this population, determine the detection rate of dysplasia in the identified lesions, and finally describe the strategies used by gastroenterologists in the region in their usual clinical practice to carry out surveillance in patients with UC.

## Materials and Methods

### Study Design and Data Collection

This is an observational, analytical, cross-sectional study, with sampling carried out “for convenience” in which the target population was adult patients with UC with a high risk of dysplasia and CRC neoplasia under control by gastroenterology in accordance with the legislation of the different LATAM countries. Patients included those monitored between January 1st and September 30th, 2023, in different Gastroenterology reference centers with experience in IBD, in Latin America. All the endoscopists have more than 10 years doing colonoscopies.

#### Inclusion criteria

Patients of legal age (>18 years) of both genders who agreed to be part of the study and signed the informed consent, with clinically quiescent UC with a duration of more than 8 years since diagnosis, or any patient with UC in remission independent of the time of diagnosis associated with the presence of primary sclerosing cholangitis. Centers with availability of the following colonoscopy techniques were qualified: High-definition white light endoscopy with random (untargeted) biopsies with performance of 4 quadrant biopsies every 10 cm (minimum 32 biopsies), endoscopy with digital chromoendoscopy NBI (Narrow Band Imaging), FICE (flexible spectral imaging color enhancement), BLI (Blue Light Imaging) or chromoendoscopy with dyes such as indigo carmine or methylene blue. We performed digital chromoendoscopy with guided selective biopsies when mucosal findings were suspicious for dysplasia or were unexplainably different from the surrounding mucosa. Dye chromoendoscopy was performed with dilute (0.03% to 0.1% indigo carmine) or absorbent (0.04% to 0.1% methylene blue) contrast dyes were liberally applied over the entire surface of the colonic mucosa during withdrawal of the colonoscope using a spray catheter.

#### Exclusion criteria

Patients with Crohn’s disease, those with active UC defined by Mayo score ≥2, poor preparation for the procedure (based on Boston score < 7), any contraindications to colonoscopy or biopsy, previous colonic surgery, or those who were scheduled for a therapeutic procedure either at the same institution or who were referred from another center with a diagnosis of dysplasia/cancer.

### Data Collection

Medical records were used as the primary source of information. Clinical variables such as gender, age, extent of IBD, age at diagnosis of IBD, pharmacological treatment for IBD, smoking history, family history of colorectal cancer, and primary sclerosing cholangitis, among others, were collected. The treatment used in each patient was recorded, including the frequency of corticosteroid requirement and the use of mesalamine. Regarding the technical aspects, the following variables were analyzed: intestinal preparation, the technology used for detection, the use of digital chromoendoscopy, and the staining method. In the endoscopic findings, the prevalence of post-inflammatory polyps was evaluated, and suspicious lesions were classified according to the Paris classification^23^Kudo,^24^ and according to the SCENIC consensus.^20^ Other variables evaluated included the resectability of the lesions, the number of biopsies, and the resection technique used. In addition, the Mayo endoscopic index and its subcomponents were evaluated,^25^ histological findings, and a frequency of non-visible dysplasia.

### Definitions

For the assessment of disease severity, the Montreal classification was considered^26^ for UC according to extent (E1: proctitis, E2: left side, and E3: extensive or pancolitis).

Response to pharmacological treatment in adults for UC was evaluated in clinical terms using the severity sub-index according to the Montreal scale (clinical remission: no symptoms of UC; mild activity: ≤4 bloody stools daily, absence of fever, pulse <90 bpm, hemoglobin ≥10.5 g/dL (105 g/L), erythrocyte sedimentation rate <30 mm/h; moderate activity: >4-5 bowel movements daily but with minimal signs of systemic toxicity; severe activity: ≥6 bloody bowel movements per day, pulse ≥90 bpm, temperature ≥99.5°F (37.5 °C), hemoglobin <10.5 g/dL (105 g/L), and erythrocyte sedimentation rate ≥30 mm/h).^26^

Patients were considered active smokers and required colorectal cancer screening by chromoendoscopy if they had a history of consumption of more than seven cigarettes per week for at least 6 months or a history of consumption of at least one cigarette in the 6 months prior to diagnosis. Patients who quit smoking at least 6 months before diagnosis were considered “ex-smokers.” “Non-smokers” were people who had never used tobacco or had used tobacco in small amounts or occasionally.^27^ Regarding personal history of PSC, it was defined as the presence of stenosis and saccular dilations of the intra- or extrahepatic bile duct preferably diagnosed with magnetic resonance cholangiopancreatography (MRCP), in patients with compatible clinical or laboratory tests, and that could not have been attributed to other causes.^28^ Regarding family history of CRC: A distinction was made between patients with a first-degree family history (parents, siblings, children) and patients with a second-degree family history (grandparents, aunts and uncles). In both groups, a distinction was made between whether the case of CRC was diagnosed before or after the age of 50 years.^29^ To assess the degree of colon cleansing, the Boston Preparedness Scale was used.^30^ Adequate preparation was defined for those with Boston global ≥6 and segments ≥2.

### Outcomes

The dysplasia lesion detection rate was defined as the total number of confirmed dysplastic lesions (of all lesions biopsied or resected) in all colonoscopies performed during this study divided by the total number of lesions detected.^31^ Dysplasia detection rate was defined as the proportion of procedures in which one or more dysplastic lesions in the colon were identified (based on the histology of a biopsy or polypectomy)^32^ divided by the total number of colonoscopies.

### Statistical Analysis

The database was developed in Excel version 2019, the processing was carried out in the social sciences program SPSS version 26.0. For the descriptive analysis of the quantitative variables, the arithmetic mean, the minimum, the maximum, and the standard deviation were used. Absolute and relative frequencies were used for qualitative variables.

To evaluate the risk factors for dysplasia, the Kolmogorov-Smirnov test was used in the bivariate analysis of quantitative variables to determine normality (*P* > .05, normality). If there was no compliance with normality, the Mann–Whitney *U* test or student’s t-test was used. If there was compliance with normality, Pearson’s Chi-square test was used for categorical variables. The multivariate analysis was determined by binary logistic regression, with the Introduce method and as a complement the backward method. For the selection of variables, the significant variables of the bivariate analysis and the theoretically important variables were taken. The OR >1 was considered as a risk factor, the OR <1 as a protective factor, and the OR equal to 1 as neutral, with a 95% confidence interval. The significance level of all tests was .05.

### Ethical Considerations

The study design took into account the requirements established in the Declaration of Helsinki, 2013 version, in Fortaleza, Brazil,^33^ and was considered a risk-free investigation. The confidentiality of patient information collected was guaranteed. This research was reviewed and approved by the institutional review board and ethics committee of each institution.

## Results

### Baseline Characteristics

A total of 144 subjects, predominantly women (55.5%), were included. All patients were recruited from different reference centers of gastroenterology and digestive endoscopy in 5 countries (Colombia, Costa Rica, Peru, Ecuador, and Dominican Republic), with a mean age of 47.3 (range 17.1 to 90; SD 15.64) years. The mean age at diagnosis of the disease was 34.58 (range 3.64 to 80.9; SD 15.45) years and the mean duration of the disease was 12.71 (range 0.64 to 57.13; SD 8.08) years (**Table 1**). As for the extent of the disease, 56.25% of patients had pancolitis, followed by left-sided colitis in 34% of patients. Also, 5.6% had a family history of colorectal cancer, and primary sclerosing cholangitis was found in 4.9% of patients. Active smoking was identified in 4.9% of patients (**Table 1**).

**Table 1.** Baseline characteristics of subjects included in the study.

Characteristics *n* = 144	
Male, *n* (%)	64 (44.4)
Age (years), mean (SD)	47.3 ± 15.64 years
Age at diagnosis, mean (SD)	34.58 ± 15.45 years
Time since diagnosis (years), mean (SD)	12.71 ± 8.08 years
**Montreal classification**	
Age at diagnosis (A1:A2: A3), n	13: 74: 57
Extension, n (E1:E2: E3)	14: 49: 81
BMI (kg/m2), mean ± SD (range)	(23.73 ± 3.63)
**Family history of CRC.**	***n*(%)**
Yes	8 (5.6)
First degree relative	3 (2.1)
Second degree relative	5 (3.5)
Diagnosed at age <50 years	2 (1.4)
Diagnosed at age >50 years	5 (3.5)
**Presence of PSC**	
PSC, *n* (%)	7 (4.9)
**Smoking status**	
Nonsmokers	126 (87.5)
Ex smokers	11 (7.6)
Active smokers	7 (4.9)

UC, ulcerative colitis; PSC, Primary sclerosing cholangitis; SD, standard deviation; BMI, body mass index; N, Number.

### Disease Activity and Medical Management

The activity index (Mayo score) was used to determine disease activity. Remission was found in most patients, with only mild activity in 38.2% of patients.

#### Treatment

A total of 36.8% had required biological or small molecule therapy, with the predominant use of anti-TNF. At the time of the study, 77.1% of patients were in management with mesalamine, 8,3% of patients with corticosteroids, and 19.4% of patients with immunomodulators. Among those using advanced therapies, 15.3% were receiving infliximab (15.3%) and 4.2% were receiving tofacitinib (**Table 2**).

**Table 2.** Medical therapy used.

*Previous medical management*	*n/ (%)*
Biologics	53 (36.8)
Anti—TNF (%)	51 (35.4)
Anti IL12-IL23	4 (2.7)
Anti-integrins	4 (2.7)
Small molecule/ tofacitinib	3 (2.1)
***Current medical treatment***	
Infliximab	22 (15.3)
Adalimumab	5 (3.5)
Golimumab	2 (1.4)
Ustekinumab	4 (2.7)
Vedolizumab	5 (3.5)
Tofacitinib	6 (4.2)
Azathioprine	28 (19.4)
Corticosteroids	12 (8.3)
Mesalamine	111 (77.1)

Anti-TNF, tumor necrosis factor inhibitors.

### Endoscopic Findings

#### Bowel preparation

In total, 71.5% of patients had adequate preparation (Boston 8 to 9) and 41% of patients had good preparation (Boston 6) (32/78).

#### Endoscopic methods

White light was used in 47.2% of the procedures and digital chromoendoscopy in 42.4% with the majority of these using NBI (20.1%). Chromoendoscopy with dye (methylene blue) was used in 9.7% (14 patients) (**Table 3**).

**Table 3.** Technical considerations.

Intestinal preparation/ Boston classification	
Poor preparation	1 (0.7%)
Good preparation	40 (27.8%)
Adequate preparation	103 (71.5%)
**Technology used**	
White light	68 (47.2%)
White light high definition	1 (0.7%)
Digital chromoendoscopy	61 (42.4%)
Chromoendoscopy with dye	14 (9.7%)
**Digital chromoendoscopy**	***n* (%)**
NBI	29 (20.1%)
LCI/BLI	27 (18.8%)
I scan	18 (12.5%)

BLI, Blue light imaging; HD, high definition; LCI, Link color imaging; NBI, Narrow band imaging; n, Number.

### Endoscopic Findings, Endoscopic Resection of Lesions, and Histological Findings

Post-inflammatory polyps were found in 33% of patients. Areas of stenosis were present in 7.6% of patients, and previous findings of dysplasia were present in 4.16% of patients.

#### Classification of lesions according to Paris

Polypoid lesions predominated (29.9%) and 2.8% (5 patients) had superficially elevated flat lesions (0-II A).

#### SCENIC consensus

It was reported by 82.5% of endoscopists. The most frequent report was disruption of the lesion edges (5.6%).

#### Kudo classification

It was reported by 90% of colonoscopists. Kudo II patients were reported in 10.4% of patients.

#### Number of biopsies

In most cases, 1-10 biopsies were taken (45.8%). Endoscopic resection of the lesions was performed in 30.6% and most (36.4%) were resected with cold snare (Table 4).

**Table 4.** Endoscopic findings, histology, and resection techniques.

Endoscopic findings	*n*/%
Post-inflammatory polyps	48 (33.3)
Strictures	11 (7.6)
**Paris classification**	
0-I	43 (29.9)
0-Ip	11 (7.6)
0-Is	32 (22.2)
0-II	5 (3.5)
0-IIa	4 (2.8)
0-IIc	1 (0.7)
0-III	1 (0.7)
Mized lesions (0-IIa + 0-II c)	1 (0.7)
***Scenic consensus***	
Lesion with ulceration	5 (3.5)
Disruption of the edges of the lesion	8 (5.6)
**Kudo classification**	
I	12 (8.3)
II	15 (10.4)
III	5 (3.5)
IV	4 (2.8)
V	3 (2.1)
**Number of biopsies**	
1-10	66 (45.8)
11-20	53 (36.8)
More than 20	14(9.7)
**Resection of lesions**	
Lesion resectability	44 (30.6)
***Technique used (n = 44)***	
Cold biopsy, n (%)	15 (34)
Cold snare polypectomy, *n* (%)	16 (36.4)
Hot snare polypectomy, *n* (%)	9 (20.4)
Endoscopic mucosal resection, *n* (%)	7 (15.9)
**Histological findings**	
Low-grade dysplasia, (%)	16 (11.1)
High-grade dysplasia, (%)	2 (1.4)
Adenocarcinoma, (%)	3 (2.1)

n, Number.

#### Histology findings

Low-grade dysplasia was present in 11.1% of patients (11/78) and adenocarcinoma was reported in three cases (2.1%) (Table 4).

All the polyps identified were resected endoscopically. Low-grade dysplasia was detected in 4 lesions of type 0-Ip, 7 lesions of type 0-Is, 3 lesions of type 0-IIa, one lesion of type 0-IIb, and one lesion of type 0-III. High-grade dysplasia was detected in one mixed lesion (0-III+IIc) and adenocarcinoma was detected in one lesion of type 0-Ip, and in two lesions of type 0-Is.

### Outcomes

In the three cases in which adenocarcinoma was detected, they underwent colectomy, and the two patients who presented findings of high-grade dysplasia were considered for follow-up since they were in long-standing remission.

#### Dysplasia was identified with random biopsies.

Non-visible dysplasia found with non-targeted biopsies was found in 3 patients (2.1%).

#### Overall rate of dysplasia detection

Dysplasia was identified in 18 patients (12.5%).

#### Dysplasia detection rate by biopsied lesion

Dysplasia was confirmed in 36.7% of the identified lesions and biopsies.

#### Risk factors for dysplasia detection

Risk analysis was performed for the detection of dysplasia in the included subjects, considering clinical information including endoscopic and histological information. In the bivariate analysis (Tables 5-8), statistical significance was identified for the variables sex, years of disease, family history of CRC, history of primary sclerosing cholangitis, history of smoking, use of biologics, endoscopic technology used for the detection of dysplasia, Kudo classification, presence of post-inflammatory polyps, histological findings and presence of dysplasia not visible in white light in random biopsies. A multivariate analysis of these was carried out.

**Table 5.** Bivariate analysis of clinical features for dysplasia detection (*n* = 144).

		Without dysplasia		With dysplasia		
		*n*	%	*n*	%	*P*
Gender	Male	37	74.00%	13	26.00%	.046
	Female	60	88.20%	8	11.80%	
Current age	Mean-SD	46.34(Mean)	15.7(SD)	51,96(Mean)	16.93(SD)	.145
Age at disease diagnosis	Mean-SD	34.83 (Mean)	16 (SD)	31,34(Mean)	14.65 (SD)	.361
Years of disease	Mean-SD	11.51(Mean)	5.45(SD)	20,61(Mean)	15.4(SD)	.004
Montreal Classification (Extent),	E1	10	90.90%	1	9.10%	.214
	E2	38	88.40%	5	11.60%	
	E3	49	76.60%	15	23.40%	
BMI	Mean-SD	23.42(Mean)	3.63(SD)	23,53(Mean)	3.35(SD)	.665
Family history of colorectal cancer	No	95	84.80%	17	15.20%	.006
	First Grade	1	33.30%	2	66.70%	
	Second Grade	1	33.30%	2	66.70%	
Age in family history of colorectal cancer	>50 years	1	20.00%	4	80.00%	NA
	< 50 years	0	0.00%	0	0.00%	
Primary sclerosing cholangitis	Yes	3	50.00%	3	50.00%	.034
	No	94	83.90%	18	16.10%	
Smoking	Nonsmoker	88	87.10%	13	12.90%	.001
	Smoker	5	71.40%	2	28.60%	
	Ex-smoker	4	40.00%	6	60.00%	

SD, standard deviation; BMI, body mass index.

**Table 6.** Bivariate analysis of therapeutic characteristics and disease activity for dysplasia detection (*n* = 144).

		Without dysplasia		With displasia		
		*n*	%	*n*	%	*P*
Use of biological therapy	Yes	25	71.40%	10	28.60%	.047
	No	72	86.70%	11	13.30%	
Biological therapies	None	1	100.00%	0	0.00%	.211
	Infliximab	13	59.10%	9	40.90%	
	Adalimunab	7	70.00%	3	30.00%	
	Golimunab	3	100.00%	0	0.00%	
	Vedolizumab	1	50.00%	1	50.00%	
	Ustekinumab	2	100.00%	0	0.00%	
	Tofacitinib	4	100.00%	0	0.00%	
Endoscopic appearance of the mucosa (Mayo Endoscopic Score)	Normal	44	81.50%	10	18.50%	.098
	Mild disease	43	89.60%	5	10.40%	
	Moderate disease	9	64.30%	5	35.70%	
	Severe disease	0	0.00%	0	0.00%	
Defecatory frequency	Normal	35	79.50%	9	20.50%	.618
	1-2 stools/day more than usual	47	87.00%	7	13.00%	
	3-4 stools/day more than usual	12	75.00%	4	25.00%	
	> 4 stools/day more than usual	3	75.00%	1	25.00%	
Mayo full score	Remission or normal	63	81.80%	14	18.20%	.052
	Mild	29	90.60%	3	9.40%	
	Moderate	5	55.60%	4	44.40%	
	Severe	0	0.00%	0	0.00%	

n, number.

**Table 7.** Bivariate analysis of endoscopic diagnostic and therapeutic technical aspects for dysplasia detection (*n* = 144).

		Without dysplasia		With dysplasia		
		*n*	%	*n*	%	*P*
Bowel preparation according to Boston scale	Bad	0	0.00%	0	0.00%	.871
	Good	20	83.30%	4	16.0%	
	Very Good	77	81.90%	17	18.10%	
Endoscopic technology for detection	White Light	62	92.50%	5	7.50%	.007
	White Light HD	1	100.00%	0	0.00%	
	Digital Chromoendoscopy	25	69.40%	11	30.60%	
	Chromoendoscopy with Staining	9	64.30%	5	35.70%	
Technology used in the case of digital chromoendoscopy	NBI	16	88.90%	2	11.10%	.056
	LCI/BLI	10	52.60%	9	47.40%	
	FICE	0	0.00%	0	0.00%	
	Iscan	8	66.70%	4	33.30%	
	SPIES	0	0.00%	0	0.00%	
Number of biopsies	0-10	40	78.40%	11	21.60%	.527
	10-20	45	86.50%	7	13.50%	
	>20	12	85.70%	2	14.30%	
Resectability of the lesion	Yes	28	63.60%	16	36.40%	.013
	No	0	0.00%	4	100.00%	
Technique of lesion resection	Cold biopsy	14	93.30%	1	6.70%	0
	Cold snare polypectomy	11	68.80%	5	31.30%	
	Hot snare polypectomy	6	66.70%	3	33.30%	
	Endoscopic Mucosal Resection	0	0.00%	7	100.00%	
	Submucosal Dissection	0	0.00%	0	0.00%	

BLI, Blue light imaging; HD, high definition; LCI, Link color imaging; NBI, Narrow band imaging; n, number; SPIES, Storz professional image enhancement system.

**Table 8.** Bivariate analysis of microscopic and macroscopic findings for dysplasia detection (*n* = 144).

		Without dysplasia		With dysplasia		
		*n*	%	*n*	%	*P*
Presence of post-inflammatory polyps	Yes	22	66.70%	11	33.30%	.006
	No	75	88.20%	10	11.80%	
Presence of stenosis	Yes	5	50.00%	5	50.00%	.005
	No	92	85.20%	16	14.80%	
Paris classification	Type: 0-Ip lesions	6	54.50%	5	45.50%	.128
	Type: 0-Is lesions	23	71.90%	9	28.10%	
	Type: 0-IIa lesions	1	25.00%	3	75.00%	
	Type: 0-IIb lesions	0	0.00%	1	100.00%	
	Type 0-IIc lesiones	0	0.00%	0	0.00%	
	Type 0-III lesiones	0	0.00%	1	100.00%	
	Mixed lesions (0-IIc+0-IIa)	0	0.00%	0	0.00%	
	Mixed lesions (0-IIa+0-IIc)	0	0.00%	1	100.00%	
	Mixed lesions (0-III+IIc u 0-IIc+III)	0	0.00%	0	0.00%	
Scenic Consensus	Ulcer	1	20.00%	4	80.00%	.279
	Disruption of lesion edges	4	50.00%	4	50.00%	
Kudo classification	Not applicable	22	91.70%	2	8.30%	0
	Kudo I	12	100.00%	0	0.00%	
	Kudo II	10	66.70%	5	33.30%	
	Kudo IIIL	0	0.00%	5	100.00%	
	Kudo IIIs	0	0.00%	4	100.00%	
	Kudo IV	0	0.00%	3	100.00%	
Histological findings	No dysplasia	97	100.00%	0	0.00%	0
	Low-grade dysplasia	0	0.00%	16	100.00%	
	High-grade dysplasia	0	0.00%	2	100.00%	
	Adenocarcinoma	0	0.00%	3	100.00%	
Presence of dysplasia not visible in white light in random biopsies.	Yes	0	0.00%	3	100.00%	0
	No	93	85.30%	16	14.70%	

n, number.

In the multivariate logistic regression analysis, the time of the disease and the presence of pseudopolyps were found to be the main risk factors for the detection of dysplasia. Regarding endoscopic methods, it was found that digital chromoendoscopy detects 4.9 times more areas of dysplasia than white light (Table 9).

**Table 9.** Multivariate analysis of associated factors for dysplasia detection.

	OR	95%CI
**Male sex**	1.7	0.53-5.63
**Years of disease**	1.1	1.01-1.21
**History of primary sclerosing cholangitis**	6.3	0.87-46.17
**Smoking**	4.1	0.64-26.30
**Requirement of biologic therapy**	1.9	0.55-6.66
**Presence of post-inflammatory polyps**	3.4	1.11-10.3
**Use of digital chromoendoscopy**	4.9	1.09-22.8
**Use of chromoendoscopy with dyes**	0.7	0.53-11.02

OR, odds ratio; 95%CI, 95% confidence interval.

## Discussion

To the best of our knowledge, this is the first study conducted in LATAM on dysplasia and CRC surveillance in UC patients. Furthermore, this is a real-world study, which is rare when evaluating UC surveillance as most studies are based on surveys, delineating the results of current practices of gastroenterologists in the region.^34^ A total of 144 patients were included, 55.6% of whom were women. The mean age of the population was 47.3 ± 15.6 years. The withdrawal time for surveillance colonoscopies recommended by experts should be 17 minutes.^35^ In the present research, this quality indicator was not provided by the participating researchers. All patients underwent the procedure under sedation, consistent with the recommendations for all patients with IBD per ECCO^36^ and other experts.^35,37–39^ Providing sedation should increase adherence to the exam in the future.^40,41^ The quality of the preparation as evaluated per Boston score was > 6 in 99% of the patients, meeting the ESGE recommendation of a minimum of 90%.^42^

Regarding the main objective of the study, dysplasia was found in 18/144 patients (12.5%) and the detection rate of dysplasia by biopsied lesion was 36.7%. These important findings warrant careful and in-depth interpretation. The rate of identification of dysplasia in surveillance colonoscopies is an important marker of quality and the European Society of Gastrointestinal Endoscopy (ESGE) considers that the minimum standard of detection of dysplasia in these patients should be 8%.^42^ In the present study, the 12.5% dysplasia detection rate is higher than recommended, as well as the 3.4% found in another real-world study^34^ and similar to the 15% (25/160) found in a recently published study from Israel in specialized IBD centers.^32^ Overall, our finding is within the range found in other studies, which ranges from 9% to 25%.^40,43–47^ This finding is encouraging and could reflect competence in different quality standards of UC surveillance colonoscopies in LATAM gastroenterologists. Some factors could be favoring this performance, such as adequate preparation of the colon, use of sedation, complete remission of the disease at the time of surveillance, reference centers, and use of 100% of high-definition endoscopes with white light and digital chromoendoscopy in 42% of patients. In this regard, it has been found that high definition, when generating images with 1 million pixels, allows one to distinguish most of the dysplasia associated with colitis^20^ and currently the use of chromoendoscopy, digital endoscopy, and high definition has a performance similar to chromoendoscopy with dyes,^18^ which facilitates the study since a simple maneuver avoids the need for preparation of the dye. Another explanation for the higher detection rate of dysplasia could be overdiagnosis, since the histology was only reviewed by one pathologist, and a second evaluation was not performed as per standard recommendations.^48^ A second opinion from another pathologist is ideal, but this is a real-world study that reflects LATAM practices where histology is reviewed by a single pathologist with experience in IBD, dysplasia, and CRC. To decrease overdiagnosis as a possibility, a prospective study would be needed including review by other expert pathologists. Interobserver concordance in low-grade dysplasia (which was the majority in this study) is poor (k = 0.41) depending on volume and years of practice.^49^

In the multivariate analysis, the risk factors associated with dysplasia were the time of disease progression and post-inflammatory polyps. Time of disease progression is a classically demonstrated variable that is associated with a higher risk of dysplasia and CRC.^8,9^ Post-inflammatory polyps had an *Odds ratio* (OR) for dysplasia of 3.4 (95% CI 1.11 to 10.3). Theoretically, this finding would be consistent with the concept of increased risk of this alteration with dysplasia, CRC, and colectomy.^50–52^ However, the 95% confidence interval is wide and could reflect an inadequate sample size. It is important to note that the positive association between post-inflammatory polyps with dysplasia and CRC is currently controversial,^8,9^ and what has more recently been deemed as a decisive factor is adequate control of the inflammatory activity.

The detection rate of dysplasia by biopsied lesion was 36.7%. The ESGE suggests that the optical diagnosis of dysplasia, verified by biopsy, should be achieved ≥10% in at least 20 lesions found with chromoendoscopy.^53^ This result is within the quality standard of optical diagnosis of dysplasia in surveillance colonoscopies in IBD and is very encouraging for the region.

This study has some limitations, including sample size, with a mixed population, which included subjects with different degrees of disease activity and different time periods of onset of UC diagnosis. Also, their methodological characteristics, without being subjected to follow-up (all were subjected to a single procedure); therefore, it is underpowered to detect specific risk factors associated with dysplasia detection or to perform subgroup analyses based on other potential risk factors associated with dysplasia. It should also be mentioned that, although it was not possible to evaluate a causal relationship between exposure and outcome, the quality of the information could also be affected by the completion of the medical records. Due to its methodological design, other outcomes of interest could not be evaluated, such as the impact of the time interval between surveillance procedures, the occurrence of metachronous lesions, or the recurrence of lesions. Also, the availability of technologies in chromoendoscopy is variable in the different countries of Latin America, many centers do not have chromoendoscopy with staining, and the sample was small to be able to make an analysis of the diagnostic performance of the different technologies. In our cases we have few patients with dye chromoendoscopy, for that, we cannot analyze this outcome significantly statistically. Finally, the different participating institutions are reference centers for patients with IBD. Therefore, patients included had more severe and complicated diseases and were followed by expert personnel in IBD. This might not be an accurate reflection of all patients with IBD in Latin American countries. However, it must be recognized that our study reflects real-world clinical practice and complexities faced on a daily basis in most Latin American countries, where clinicians manage a diverse patient population with uneven access to healthcare and limited resources given socioeconomic, cultural, educational, and geographical barriers. This study provides an initial overview of the efforts that have been made in these countries to raise awareness of IBD among the Latino population and the impact of cancer surveillance on these patients. There is an urgent need for education and accurate population data given the exponential rise in the incidence and prevalence of IBD in Latin America and the obvious differences between this population and other IBD populations in Europe, Asia, and North America.

## Conclusions

This study shows encouraging results in dysplasia detection rates, suggesting that the implementation of a specific surveillance program in colonoscopy in IBD is an effective strategy for diagnosing dysplastic lesions with high detection rates. In our region, endoscopic techniques with digital chromoendoscopy with targeted biopsies are preferred. Most lesions were endoscopically visible and resectable suggesting that digital chromoendoscopy with targeted biopsies is an effective strategy compared to other techniques. Improving compliance with quality indicators in colonoscopy for endoscopic follow-up of patients with IBD is a goal to be implemented in surveillance programs. Further research should be continued and encouraged in Latin American countries to define optimal values for dysplasia detection rate in patients with IBD and high risk, and clinical differences between these patients and what has been previously reported to ensure adequate management and surveillance.

## Data Availability

The data that support the findings of this study are available on request from the corresponding author, VPI and JSFO. The data are not publicly available due to privacy/ethical restrictions, containing information that could compromise the privacy of research participants.

## References

[1] Gros B , Kaplan GG. Ulcerative colitis in adults: a review. JAMA. 2023;330(10):951-965. doi: https://doi.org/10.1001/jama.2023.15389 37698559

[2] Patil DT , Odze RD. Backwash is hogwash: the clinical significance of ileitis in ulcerative colitis. Am J Gastroenterol. 2017;112(8):1211-1214. doi: https://doi.org/10.1038/ajg.2017.182 28631729

[3] Hoentjen F , Dieleman LA. Pathophysiology of inflammatory bowel diseases. In: Gibson GR, Roberfroid M, eds. Handbook of Prebiotics. 1st ed. Vol 1. CRC Press; 2008:341-374. doi:10.1201/9780849381829-22

[4] Gordon IO , Agrawal N, Willis E, et al Fibrosis in ulcerative colitis is directly linked to severity and chronicity of mucosal inflammation. Aliment Pharmacol Ther. 2018;47(7):922-939. doi: https://doi.org/10.1111/apt.14526 29411405 PMC5842117

[5] Raine T , Bonovas S, Burisch J, et al ECCO guidelines on therapeutics in ulcerative colitis: medical treatment. J Crohns Colitis. 2022;16(1):2-17. doi: https://doi.org/10.1093/ecco-jcc/jjab178 34635919

[6] Rogler G. Chronic ulcerative colitis and colorectal cancer. Cancer Lett. 2014;345(2):235-241. doi: https://doi.org/10.1016/j.canlet.2013.07.032 23941831

[7] Jess T , Rungoe C, Peyrin-Biroulet L. Risk of colorectal cancer in patients with ulcerative colitis: a meta-analysis of population-based cohort studies. Clin Gastroenterol Hepatol. 2012;10(6):639-645. doi: https://doi.org/10.1016/j.cgh.2012.01.010 22289873

[8] Mahmoud R , Shah SC, ten Hove JR, et al; Dutch Initiative on Crohn and Colitis. No association between pseudopolyps and colorectal neoplasia in patients with inflammatory bowel diseases. Gastroenterology. 2019;156(5):1333-1344.e3. doi: https://doi.org/10.1053/j.gastro.2018.11.067 30529584 PMC7354096

[9] Jong ME , Gillis VELM, Derikx LAAP, Hoentjen F. No increased risk of colorectal neoplasia in patients with inflammatory bowel disease and postinflammatory polyps. Inflamm Bowel Dis. 2020;26(9):1383-1389. doi: https://doi.org/10.1093/ibd/izz261 31677385 PMC7441099

[10] Gordon H , Biancone L, Fiorino G, et al ECCO guidelines on inflammatory bowel disease and malignancies. J Crohns Colitis. 2023;17(6):827-854. doi: https://doi.org/10.1093/ecco-jcc/jjac187 36528797

[11] Eaden JA , Abrams KR, Mayberry JF. The risk of colorectal cancer in ulcerative colitis: a meta-analysis. Gut. 2001;48(4):526-535. doi: https://doi.org/10.1136/gut.48.4.526 11247898 PMC1728259

[12] Seishima R , Okabayashi K, Ikeuchi H, et al Effect of biologics on the risk of advanced-stage inflammatory bowel disease-associated intestinal cancer: a nationwide study. Am J Gastroenterol. 2023;118(7):1248-1255. doi: https://doi.org/10.14309/ajg.0000000000002149 36622356

[13] Dixon SW , Collard TJ, Mortensson EMH, et al 5-Aminosalicylic acid inhibits stem cell function in human adenoma-derived cells: implications for chemoprophylaxis in colorectal tumorigenesis. Br J Cancer. 2021;124(12):1959-1969. doi: https://doi.org/10.1038/s41416-021-01354-5 33785874 PMC8184823

[14] Shah SC , Itzkowitz SH. Colorectal cancer in inflammatory bowel disease: mechanisms and management. Gastroenterology. 2022;162(3):715-730.e3. doi: https://doi.org/10.1053/j.gastro.2021.10.035 34757143 PMC9003896

[15] Bye WA , Ma C, Nguyen TM, Parker CE, Jairath V, East JE. Strategies for detecting colorectal cancer in patients with inflammatory bowel disease: a cochrane systematic review and meta-analysis. Am J Gastroenterol. 2018;113(12):1801-1809. doi: https://doi.org/10.1038/s41395-018-0354-7 30353058 PMC6768584

[16] Wijnands AM , de Jong ME, Lutgens MWMD, Hoentjen F, Elias SG, Oldenburg B; Dutch Initiative on Crohn and Colitis (ICC). Prognostic factors for advanced colorectal neoplasia in inflammatory bowel disease: systematic review and meta-analysis. Gastroenterology. 2021;160(5):1584-1598. doi: https://doi.org/10.1053/j.gastro.2020.12.036 33385426

[17] Lamb CA , Kennedy NA, Raine T, et al; IBD guidelines eDelphi consensus group. British society of gastroenterology consensus guidelines on the management of inflammatory bowel disease in adults. Gut. 2019;68(Suppl 3):s1-s106. doi: https://doi.org/10.1136/gutjnl-2019-318484 31562236 PMC6872448

[18] Murthy SK , Feuerstein JD, Nguyen GC, Velayos FS. AGA clinical practice update on endoscopic surveillance and management of colorectal dysplasia in inflammatory bowel diseases: expert review. Gastroenterology. 2021;161(3):1043-1051.e4. doi: https://doi.org/10.1053/j.gastro.2021.05.063 34416977

[19] Shergill AK , Farraye FA. Toward a consensus on endoscopic surveillance of patients with colonic inflammatory bowel disease. Gastrointest Endosc Clin N Am. 2014;24(3):469-481. doi: https://doi.org/10.1016/j.giec.2014.03.006 24975537

[20] Laine L , Kaltenbach T, Barkun A, McQuaid KR, Subramanian V, Soetikno R; SCENIC Guideline Development Panel. SCENIC international consensus statement on surveillance and management of dysplasia in inflammatory bowel disease. Gastroenterology. 2015;148(3):639-651.e28. doi: https://doi.org/10.1053/j.gastro.2015.01.031 25702852

[21] Galanopoulos M , Tsoukali E, Gkeros F, Vraka M, Karampekos G, Matzaris GJ. Screening and surveillance methods for dysplasia in inflammatory bowel disease patients: where do we stand? World J Gastrointest Endosc. 2018;10(10):250-258. doi: https://doi.org/10.4253/wjge.v10.i10.250 30364842 PMC6198309

[22] Klepp P , Tollisen A, Røseth A, et al Real-life chromoendoscopy for dysplasia surveillance in ulcerative colitis. World J Gastroenterol. 2018;24(35):4069-4076. doi: https://doi.org/10.3748/wjg.v24.i35.4069 30254411 PMC6148427

[23] The Paris endoscopic classification of superficial neoplastic lesions: Esophagus, stomach, and colon - November 30 to December 1, 2002. Gastrointest Endosc. 2003;58(6 Suppl):S3-S43. doi: https://doi.org/10.1016/s0016-5107(03)02159-x 14652541

[24] Kudo SE , Tamura S, Nakajima T, Yamano HO, Kusaka H, Watanabe H. Diagnosis of colorectal tumorous lesions by magnifying endoscopy. Gastrointest Endosc. 1996;44(1):8-14. doi: https://doi.org/10.1016/s0016-5107(96)70222-5 8836710

[25] Schroeder KW , Tremaine WJ, Ilstrup DM. Coated oral 5-aminosalicylic acid therapy for mildly to moderately active ulcerative colitis. N Engl J Med. 1987;317(26):1625-1629. doi: https://doi.org/10.1056/NEJM198712243172603 3317057

[26] Gasche C , Scholmerich J, Brynskov J, et al A simple classification of Crohn’s disease: report of the working party for the world congresses of gastroenterology, Vienna 1998. Inflamm Bowel Dis. 2000;6(1):8-15. doi: https://doi.org/10.1097/00054725-200002000-00002 10701144

[27] Nunes T , Etchevers MJ, Merino O, et al High smoking cessation rate in Crohn’s disease patients after physician advice - The TABACROHN Study. J Crohns Colitis. 2013;7(3):202-207. doi: https://doi.org/10.1016/j.crohns.2012.04.011 22626507

[28] Angulo P , Lindor KD. Primary sclerosing cholangitis. Hepatology. 1999;30(1):325-332. doi: https://doi.org/10.1002/hep.510300101 10385674

[29] Taylor DP , Burt RW, Williams MS, Haug PJ, Cannon-Albright LA. Population-based family history-specific risks for colorectal cancer: a constellation approach. Gastroenterology. 2010;138(3):877-885. doi: https://doi.org/10.1053/j.gastro.2009.11.044 19932107 PMC2831153

[30] Lai EJ , Calderwood AH, Doros G, Fix OK, Jacobson BC. The Boston bowel preparation scale: a valid and reliable instrument for colonoscopy-oriented research. Gastrointest Endosc. 2009;69(3 Pt 2):620-625. doi: https://doi.org/10.1016/j.gie.2008.05.057 19136102 PMC2763922

[31] Bossard C , Denis MG, Bézieau S, et al Involvement of the serrated neoplasia pathway in inflammatory bowel disease-related colorectal oncogenesis. Oncol Rep. 2007;18(5):1093-1097 17914558

[32] Snir Y , Ollech JE, Peleg N, et al Dysplasia detection rates under a surveillance program in a tertiary referral center for inflammatory bowel diseases: Real-world data. Dig Liver Dis. 2023;56(2):265-271. doi: https://doi.org/10.1016/j.dld.2023.10.001 37858514

[33] World medical association declaration of Helsinki: ethical principles for medical research involving human subjects. JAMA. 2013;310(20):2191-2194. doi: https://doi.org/10.1001/jama.2013.281053 24141714

[34] Elford AT , Hirsch R, McKay OM, et al Identifying the real-world challenges of dysplasia surveillance in inflammatory bowel disease: a retrospective cohort study in a tertiary health network. Intern Med J. 2024;54(1):96-103. doi: https://doi.org/10.1111/imj.16102 37093665

[35] Zilli A , Capogreco A, Furfaro F, et al Improving quality of care in endoscopy of inflammatory bowel disease: can we do better? Expert Rev Gastroenterol Hepatol. 2020;14(9):819-828. doi: https://doi.org/10.1080/17474124.2020.1780913 32543983

[36] Fiorino G , Lytras T, Younge L, et al Quality of care standards in inflammatory bowel diseases: a European Crohn’s and colitis organisation [ECCO] position paper. J Crohns Colitis. 2020;14(8):1037-1048. doi: https://doi.org/10.1093/ecco-jcc/jjaa023 32032423

[37] Denters MJ , Schreuder M, Depla ACTM, et al Patients’ perception of colonoscopy: Patients with inflammatory bowel disease and irritable bowel syndrome experience the largest burden. Eur J Gastroenterol Hepatol. 2013;25(9):964-972. doi: https://doi.org/10.1097/MEG.0b013e328361dcd3 23660935

[38] Buisson A , Gonzalez F, Poullenot F, et al; ACCEPT study group. Comparative acceptability and perceived clinical utility of monitoring tools: a nationwide survey of patients with inflammatory bowel disease. Inflamm Bowel Dis. 2017;23(8):1425-1433. doi: https://doi.org/10.1097/MIB.0000000000001140 28570431

[39] Christensen KR , Steenholdt C, Buhl S, Brynskov J, Ainsworth MA. Tu1006 – development of an evidence-based strategy incorporating patient reported outcomes and physicians’ preferences to monitor biological therapies in inflammatory bowel disease. Gastroenterology. 2019;156(19):S-943. doi: https://doi.org/10.1016/s0016-5085(19)39319-9

[40] Efthymiou M , Allen PB, Taylor ACF, et al Chromoendoscopy versus narrow band imaging for colonic surveillance in inflammatory bowel disease. Inflamm Bowel Dis. 2013;19(10):2132-2138. doi: https://doi.org/10.1097/MIB.0b013e31829637b9 23899540

[41] Bisschops R , Bessissow T, Joseph JA, et al Chromoendoscopy versus narrow band imaging in UC: a prospective randomised controlled trial. Gut. 2018;67(6):1087-1094. doi: https://doi.org/10.1136/gutjnl-2016-313213 28698230

[42] Dekker E , Nass KJ, Iacucci M, et al Performance measures for colonoscopy in inflammatory bowel disease patients: European Society of Gastrointestinal Endoscopy (ESGE) quality improvement initiative. Endoscopy. 2022;54(9):904-915. doi: https://doi.org/10.1055/a-1874-0946 35913069

[43] Picco MF , Pasha S, Leighton JA, et al Procedure time and the determination of polypoid abnormalities with experience: implementation of a chromoendoscopy program for surveillance colonoscopy for ulcerative colitis. Inflamm Bowel Dis. 2013;19(9):1913-1920. doi: https://doi.org/10.1097/MIB.0b013e3182902aba 23811635

[44] Iacucci M , Hassan C, Gasia MF, et al Serrated adenoma prevalence in inflammatory bowel disease surveillance colonoscopy, and characteristics revealed by chromoendoscopy and virtual chromoendoscopy. Can J Gastroenterol Hepatol. 2014;28(11):589-594. doi: https://doi.org/10.1155/2014/386540 25575106 PMC4277170

[45] Gasia MF , Ghosh S, Panaccione R, et al Targeted biopsies identify larger proportions of patients with colonic neoplasia undergoing high-definition colonoscopy, dye chromoendoscopy, or electronic virtual chromoendoscopy. Clin Gastroenterol Hepatol. 2016;14(5):704-712. doi: https://doi.org/10.1016/j.cgh.2015.12.047 26804384

[46] Carballal S , Maisterra S, López-Serrano A, et al; EndoCAR group of the Spanish Gastroenterological Association and Spanish Digestive Endoscopy Society. Real-life chromoendoscopy for neoplasia detection and characterisation in long-standing IBD. Gut. 2018;67(1):70-78. doi: https://doi.org/10.1136/gutjnl-2016-312332 27612488

[47] Moussata D , Allez M, Cazals-Hatem D, et al; the GETAID. Are random biopsies still useful for the detection of neoplasia in patients with IBD undergoing surveillance colonoscopy with chromoendoscopy? Gut. 2018;67(4):616-624. doi: https://doi.org/10.1136/gutjnl-2016-311892 28115492

[48] Rubin DT , Ananthakrishnan AN, Siegel CA, Sauer BG, Long MD. ACG clinical guideline: ulcerative colitis in adults. Am J Gastroenterol. 2019;114(3):384-413. doi: https://doi.org/10.14309/ajg.0000000000000152 30840605

[49] Jimeno M , Domingo A, Salas I, et al Pathologist experience and concordance in the diagnosis of dysplasia in long-standing inflammatory bowel disease. Am J Surg Pathol. 2020;44(7):955-961. doi: https://doi.org/10.1097/PAS.0000000000001475 32235151

[50] Velayos FS , Loftus EV, Jess T, et al Predictive and protective factors associated with colorectal cancer in ulcerative colitis: a case-control study. Gastroenterology. 2006;130(7):1941-1949. doi: https://doi.org/10.1053/j.gastro.2006.03.028 16762617

[51] Rutter MD , Saunders BP, Wilkinson KH, et al Cancer surveillance in longstandinq ulcerative colitis: endoscopic appearances help predict cancer risk. Gut. 2004;53(12):1813-1816. doi: https://doi.org/10.1136/gut.2003.038505 15542520 PMC1774334

[52] Feakins RM ; British Society of Gastroenterology. Inflammatory bowel disease biopsies: updated British society of gastroenterology reporting guidelines. J Clin Pathol. 2013;66(12):1005-1026. doi: https://doi.org/10.1136/jclinpath-2013-201885 23999270

[53] Houwen BBSL , Dekker E, Puig I, et al Curriculum for optical diagnosis training in Europe: European Society of Gastrointestinal Endoscopy (ESGE) position statement. Endoscopy. 2020;52(10):899-923. doi: https://doi.org/10.1055/a-1231-5123 32882737

